# *Clostridium difficile*-associated diarrhoea in a tertiary referral neurocritical care centre

**DOI:** 10.1186/cc9651

**Published:** 2011-03-11

**Authors:** S Tripathy, C Kataria, PV Nair

**Affiliations:** 1 Walton Centre of Neurosciences, Liverpool, UK

## Introduction

*Clostridium difficile*-associated diarrhoea (CDAD) is associated with a mortality of up to 25% in susceptible patients. It occurs following long-term hospitalisation and prolonged antibiotic usage, particularly cephalosporins. Neurointensive care unit (NICU) patients on average have higher bed days, greater incidence of ventilator-associated pneumonia (VAP) and higher antibiotic use. We aimed to study the aetiology, acquisition rate and outcome of NICU-acquired CDAD.

## Methods

Intensive care admission and hospital infection control databases from April 2008 to August 2010 were studied and the case notes reviewed retrospectively. Patients who acquired CDAD within 48 hours of NICU admission were excluded. Diarrhoea was classified as mild, moderate or severe based on frequency and volume. Information on use of antibiotics, frequency, duration and type was gathered. Admission diagnosis, days of NICU stay and incidence of complications were noted.

## Results

Of the 2,212 patients with a total of 10,825 bed-days, nine patients developed CDAD. The mean NICU stay was 26 (11 to 103) days. The median duration between ICU admission and development of CDAD was 11 (3 to 93) days (7 in other neurocritical care units). Median age of the patients was 55 (20 to 72) years. Patients had a mean 6.7 (±5.2) days of diarrhoea prior to a positive assay. At the time of diagnosis, four (44%) patients had moderate disease. Three patients had a perceived delay in discharge from the ICU (1 to 8 days) due to their infective status. Concurrent infections occurred in 77% of patients, 33% of which were VAP. Of the antibiotics used prior to CDAD diagnosis, 44% were cephalosporins. There were no major complications or mortality attributed to CDAD. Identified risk factors for ICU-acquired CDAD included age >65 (22%), antibiotics (67%), laxatives (100%), steroids (33%), proton pump inhibitors (88%) and medical device requirement (100%). All patients were emergency admissions, of which eight were neurosurgical. The one patient who had the most protracted disease was isolated with *C. difficile *ribotype 027.

## Conclusions

In spite of a patient population who is at high risk of CDAD, the rate of infection in our unit is 8.3 per 10,000 bed-days or 0.4% incidence, which is below the average incidence for general intensive care (10.6 per 10,000 bed-days) and neurocritical care units (0.6%) in the UK. This may be attributed to the presence of an efficient infection control team, isolation practices with patients immediately being isolated to barrier nursing and a protocol for CDAD detection as well as a high degree of awareness amongst the medical and nursing staff.

